# Changes in the respiratory microbiome during acute exacerbations of idiopathic pulmonary fibrosis

**DOI:** 10.1186/s12931-017-0511-3

**Published:** 2017-02-01

**Authors:** Philip L. Molyneaux, Michael J Cox, Athol U. Wells, Ho Cheol Kim, Wonjun Ji, William O. C. Cookson, Miriam F. Moffatt, Dong Soon Kim, Toby M. Maher

**Affiliations:** 1 National Heart and Lung Institute, Imperial College, London, UK; 2 Royal Brompton Hospital, London, UK; 3 Asan Medical Center, University of Ulsan, Seoul, Korea; 4 Fibrosis Research Group, Inflammation, Repair & Development Section, NHLI, Sir Alexander Fleming Building, Imperial College, London, SW7 2AZ UK

**Keywords:** Idiopathic pulmonary fibrosis, Acute exacerbation, Bacterial infection, 16S

## Abstract

Acute exacerbations of idiopathic pulmonary fibrosis (AE-IPF) have been defined as events of clinically significant respiratory deterioration with an unidentifiable cause. They carry a significant mortality and morbidity and while their exact pathogenesis remains unclear, the possibility remains that hidden infection may play a role. The aim of this pilot study was to determine whether changes in the respiratory microbiota occur during an AE-IPF. Bacterial DNA was extracted from bronchoalveolar lavage from patients with stable IPF and those experiencing an AE-IPF. A hyper-variable region of the 16S ribosomal RNA gene (16S rRNA) was amplified, quantified and pyrosequenced. Culture independent techniques demonstrate AE-IPF is associated with an increased BAL bacterial burden compared to stable disease and highlight shifts in the composition of the respiratory microbiota during an AE-IPF.

## Background

Current diagnostic criteria for acute exacerbations of idiopathic pulmonary fibrosis (AE-IPF) clearly distinguish them from exacerbations of other respiratory diseases [1]. Prior to the latest iteration of the guidelines diagnosis of an AE-IPF specifically required the exclusion of any infective trigger [2]. The exact pathogenesis, however, remains unknown and it is currently unclear whether AE-IPF represent an accelerated phase of the underlying fibroproliferative process or an exaggerated lung injury response to unidentified preceding or coexistent infection [3]. There are a number of factors supporting a role for infection; i) seasonal patterns exist with increased AE-IPF occurring during the winter months, ii) respiratory tract infections in individuals with IPF confer a mortality risk indistinguishable from that seen with acute exacerbations [4], iii) *post mortem* examination in cases of confirmed infection frequently discloses diffuse alveolar damage identical to that seen during an AE-IPF and, iv) immunosuppression is associated with an increased rate of acute exacerbations [5].

Given the limited sensitivity of culture-dependant clinical microbiology techniques it is plausible that many episodes of apparent AE-IPF arise as the sequelae of infection [6]. While the role of viruses in AE-IPF has been extensively investigated [7] there has been little focus on a potential role for bacteria, despite recent evidence that bacterial burden is increased in the IPF lung and changes in the microbiome relate to disease outcomes [8, 9]. In order to explore changes in the bronchoalveolar lavage (BAL) microbiota during an AE-IPF, 16S rRNA gene qPCR and pyrosequencing were performed on both stable and exacerbation samples in subjects with IPF.

## Materials

### Study design

Patients with AE-IPF undergoing bronchoscopy were recruited prospectively from the University of Ulsan, Korea. A diagnosis of IPF was made, according to international guidelines [10], following multidisciplinary team discussion. A diagnosis of an acute exacerbation was made based on the established criteria at that time [2]. All subjects with acute exacerbation had negative clinical evaluation for infection including; respiratory syncytial virus, influenza A and B, human parainfluenza viruses, adenovirus, human cytomegalovirus, herpes simplex, varicella-zoster virus and standard bacterial culture. Control patients with stable IPF (defined by the absence of acute exacerbation) were recruited alongside AE-IPF patients and underwent bronchoscopy at the time of diagnosis. All bronchoscopies were performed as part of routine clinical care within the first 48 h of admission and ideally before the commencement of any antibiotics. However, a number of AE-IPF subjects did receive antibiotics before lavage. Written informed consent was obtained from all subjects and the study received approval from the local institutional review board.

### Bronchoscopy

Fibre-optic bronchoscopy with BAL was performed as part of routine clinical evaluation, and in subjects undergoing AE-IPF was undertaken within 48 h of admission to hospital. Bronchoscopy was performed according to a standard operating procedure [11] and BAL was performed in a single sub-segment of the right middle lobe, with at least 100 ml of sterile saline instilled. BAL fluid was examined for the presence of macrophages to confirm access of the alveolar compartment, and the absence of ciliated epithelium was used to exclude large airway contamination.

### DNA extraction

The samples were processed at the time of collection and subsequently stored at -80 °C as separate cell pellets and supernatants. The samples, one 2 ml aliquot of BAL supernatant and one BAL pellet including supernatant, were thawed and then centrifuged at 20,000 g for 15 min, to pellet cell debris and bacteria. Each pellet was then re-suspended and combined allowing genomic DNA to be isolated using the MP Bio FastDNA® SPIN Kit for Soil (http://www.mpbio.com) as previously described [12].

### 16S rRNA gene qPCR and Pyrosequencing

The V3-V5 region of the bacterial 16S rRNA gene was amplified using the 357 F forward primer and the 926R reverse primer for both 16S rRNA gene qPCR and pyrosequencing as previously described [12].

### Statistical analysis

Analysis was undertaken as described previously [8] with continuous variables presented as means (± Standard Deviation [SD]), and categorical variables as proportions. Metastats was used to perform non-parametric *t*-test comparisons of microbial communities between groups, with *P*-values corrected by multiple hypotheses testing using the FDR approach of Benjamini and Hochberg [13]. We restricted testing to Operational Taxonomic Units (OTUs) that had a differing mean abundance between cases and controls of more than 1% of the total. Shannon’s entropy [14] (alpha diversity index) and weighted and unweighted UniFrac distances [15] (beta diversity) were calculated in QIIME. Differences between subject groups were evaluated with the use of the Mann–Whitney test for continuous variables and Fisher’s exact test for categorical variables. The statistical significance of association of variables with an AE-IPF was assessed using a stepwise backward elimination logistic regression process to select among potential covariates for inclusion in the final model. All analyses were performed with the use of SPSS (version 21) and R (http://cran.r-project.org/). A two-sided *P* value of less than 0.05 was considered to indicate statistical significance.

## Results

Twenty patients with acute exacerbations of IPF and 15 matched control patients with stable IPF were enrolled at the University of Ulsan in Seoul, Korea (Table 1) [7]. The patients were matched for age, sex, smoking history and baseline lung function. There was no significant difference in the use of immunosuppressive therapy between groups. Patients had negative lavage bacterial culture and respiratory virus screens. There was no significant difference in lavage cell count between either group. There was a significantly higher neutrophil count in the lavage of AE-IPF subjects compared to controls (23.7 ± 4.39 vs. 9.0 ± 3.27; *P =* 0.037) that occurred at the expense of monocytes (40.9 ± 3.73 vs. 70.3 ± 3.73; *P =* <0.005). There was no significant difference in the lymphocyte, basophil or eosinophil cell populations. The average survival in the AE-IPF cohort was 187 (±65) days compared to 568 (±70) days in the controls (*P* = 0.0004). Thirteen (65%) of the subjects experiencing an exacerbation died in the following 60 days and six patients received mechanical ventilation.

**Table 1:** Baseline Characteristics of the Subjects

	Stable IPF (*N* = 15)	AE-IPF (*N* = 20)
Age (yr)	66.7 (±6.4)	66.3 (±6.7)
Female Sex - number (%)	3 (20%)	5 (25%)
Smoking (Ever v Never) - number (%)	11 (73%)	15 (75%)
FVC - %	79.0 (±21)	80.0 (±19)
DLCO - %	69.0 (±14)	66.0 (±16)

Details are provided for stable IPF and for AE-IPF cases. All lung function data was recorded when stable. There were no significant differences between either cohort. Data are mean ± Standard Deviation. DLCO, carbon monoxide diffusion capacity; FVC, forced vital capacity

All 35 samples yielded genomic DNA and underwent amplification of the V3-V5 region of the 16S rRNA gene. Three of the samples (two exacerbation samples and one stable sample) yielded insufficient 16S rRNA gene product to undergo pyrosequencing and were therefore excluded. All of the samples in this study were extracted, amplified and sequenced together in a single batch, to control for the potential effects of batch and operator variability. Sequence data has been submitted to the European Nucleotide Archive and is available under accession number PRJEB18649 (http://www.ebi.ac.uk/ena/data/view/PRJEB18649). In addition the denoised reads, OTU table and clinical data are available to download from the EMBL-EBI BioStudies database under accession number S-BSST12 (https://www.ebi.ac.uk/biostudies/studies/S-BSST12).

**Fig. 1:**
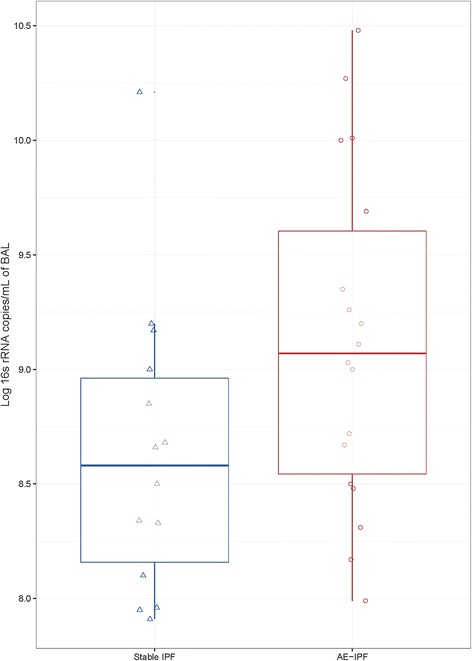
Bacterial load in acute exacerbation of IPF compared with stable disease

AE-IPF subjects had on average 4.9×10^9^ (SD ± 7.9 × 10^9^) copies of the 16S rRNA gene per ml of BAL. This was over four times higher than the copy number in the stable IPF subjects (*P* = 0.012) (Fig. 1). Negative control samples yielded a bacterial burden close to or below the lower limit of qPCR quantification (1,000 copies/ml). Bacterial burden remained significantly associated with a diagnosis of AE-IPF, using a stepwise logistic regression incorporating sex, age and smoking status (*P* = 0.029, R^2^ 0.51) and correlated strongly with disease state (Spearman’s ρ = 0.45, *P =* 0.008), indicating that total bacterial load is significantly associated with an AE-IPF.

**Fig. 2:**
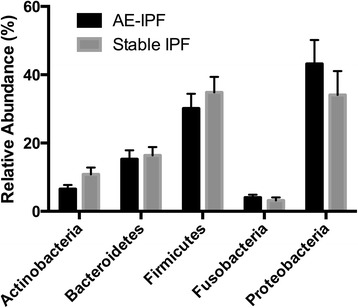
Changes in specific bacterial species in acute exacerbations of IPF compared with stable disease

At phylum level the microbiota of the stable IPF subjects was dominated by Firmicutes (34%), Proteobacteria (32%), Bacteroidetes (16%) and Actinobacteria (10%). Although the same phyla predominated in the AE-IPF subjects, Proteobacteria accounted for over 40% of the total reads (*P* = 0.12), with the percentage of reads assigned to Firmicutes, Bacteroidetes, and Actinobacteria dropping compared to the stable subjects (29, 14 and 6% respectively) (Fig. 2). Comparison at an OTU level identified that within the Proteobacteria phylum there was a higher relative abundance of two potentially pathogenic OTUs in the AE-IPF samples; *Campylobacter sp.* (*P* = 0.02) and *Stenotrophomonas sp.* (*P* = 0.03). By contrast, significantly higher numbers of a *Veillonella sp.* (*P* < 0.01) were present in the stable IPF samples. Han and colleagues [16] found both *Streptococcus* and *Staphylococcus* OTUs to be associated with IPF disease progression. Here we do not find any significant difference in the proportion of either species between the stable and exacerbation groups.

## Discussion

AE-IPF is associated with an increased BAL bacterial burden compared to stable IPF. The bacterial communities of the stable Korean IPF subjects were found to contain *Streptococcus, Prevotella, Veillonella, Haemophilus* and *Pseudomonas* which are all commonly found in the airways of healthy individuals as well as subjects with asthma, COPD and IPF [8, 9, 12, 17, 18]. Following an AE-IPF there was a notable change in the microbiota with an increase in two potentially pathogenic Proteobacterial OTUs; *Campylobacter* sp*.* and *Stenotrophomonas* sp., coupled with a significant decrease in *Veillonella* sp*.*.

*Campylobacter*, although best known as a gastrointestinal pathogen, has previously been identified in the respiratory microbiota of individuals with severe COPD. Its presence in the respiratory microbiota is likely to arise from silent micro-aspiration of gastric contents and strengthens the already identified association of reflux and aspiration with acute exacerbations in a subgroup of patients [19]. *Stenotrophomonas*, a Gram-negative bacterium, is known to colonise the respiratory tract of patients with chronic lung disease [18]. It is a potential respiratory pathogen and although most commonly associated with intubated and ventilated patients, it is responsible for respiratory infections in cystic patients and patients with chronic health conditions.

It is important to note that there are a number of limitations in relation to this study. The very nature of AE-IPF make them severe and unpredictable events and therefore microbiological sampling is often difficult and carries a high morbidity. This is evident by the fact that six out of 20 AE-IPF subjects in the current study were intubated at the time of sampling, however, when the data here were analyzed with these subjects excluded, the observed differences remained unchanged. These patients were prospectively recruited and sampled, however, the microbiome work was retrospective and bronchoscopy samples were collected out of clinical necessity within the first 24 h of admission. This was ideally before the commencement of any antibiotics, however, a number of AE-IPF subjects did receive antibiotics therapy prior to sampling. Despite this antibiotic therapy there was a significant increase in the bacterial burden and changes in the microbiota, suggesting that antibiotic therapy does not completely confound the observed differences associated with exacerbation, as it might be expected that antibiotics would reduce the bacterial burden. Future studies will have to concentrate on overcoming the challenge of prospective sampling; something which has yet to be achieved in this rapidly progressive and often fatal complication of IPF. However, until we understand the aetiology of these events in more detail the clinical urge will remain to start empiric anti-microbial therapy at the earliest opportunity when managing a patient with an AE-IPF.

There are currently no precise methods of ruling out upper airway bronchoscopic carry over of bacteria, even with upper airway samples [20]. In this study the same protocols, procedures and kit batches were used for stable and AE-IPF samples, rendering any potential contamination consistent across comparative groups and therefore unlikely to have influenced the results. Specifically none of the OTUs demonstrated a correlation with a lower bacterial burden, which can indicate a contaminant [21] and the inclusion of negative reagent controls, which yielded bacterial burden close to or below the lower limit of qPCR quantification, also demonstrates that the qPCR results are not a sampling or processing artefact.

Unlike exacerbations of other respiratory conditions which are truly acute events, the onset of an exacerbation in IPF is more insidious with a gradual worsening over days to weeks. Consequently by the time of presentation any triggering viruses may no longer be detectable, despite exhaustive searching [22]. Viruses have been shown to impact the respiratory microbiome, causing changes which persist for weeks, so the differences identified could represent the consequence of an unidentified preceding viral insult [12].

## Conclusions

In summary differences in specific OTUs and bacterial burden suggest that bacteria may play a causative role in some AE-IPF. The apparent translocation of bacteria usually confined to the gastrointestinal tract also suggests a role for aspiration in the development of acute exacerbations. This pilot study had a number of limitations and a prospective study with paired samples prior to and during an exacerbation will be required to test the hypotheses generated by this data. Nonetheless, our observations challenge the current paradigm for AE-IPF and provide a rationale for clinical trials of prophylactic antibiotics as a strategy to prevent acute exacerbations in individuals with IPF.

## Abbreviations

16S rRNA: 16S ribosomal RNA gene
AE-IPF: Acute exacerbation of IPF
BAL: Bronchoalveolar lavage
OTUs: Operational Taxonomic Units
